# Real-World Effectiveness of Vedolizumab Dose Escalation in Patients With Inflammatory Bowel Disease: A Systematic Literature Review

**DOI:** 10.1093/crocol/otac020

**Published:** 2022-07-08

**Authors:** Dipen Patel, Stephan Martin, Michelle Luo, Lyann Ursos, Richard A Lirio, Pravin Kamble, Song Wang

**Affiliations:** Open Health, Bethesda, Massachusetts, USA; Open Health, Bethesda, Massachusetts, USA; Takeda, Cambridge, Massachusetts, USA; Takeda Pharmaceuticals USA Inc., Deerfield, Illinois, USA; Takeda, Cambridge, Massachusetts, USA; Takeda, Cambridge, Massachusetts, USA; Takeda, Cambridge, Massachusetts, USA

**Keywords:** vedolizumab, ulcerative colitis, Crohn’s disease, dose escalation

## Abstract

**Background:**

Vedolizumab is a gut-selective anti-lymphocyte trafficking agent approved for the treatment of moderate to severely active inflammatory bowel disease (IBD: ulcerative colitis [UC] and Crohn’s disease [CD]).

**Methods:**

A systematic literature review (SLR) of real-world studies was conducted to assess the effectiveness of dose escalation of vedolizumab every 8 weeks (Q8W) during maintenance treatment to achieve a response in patients who were either vedolizumab responders experiencing secondary loss of response (SLOR) or non-responders. MEDLINE and EMBASE databases were searched from January 2014 to August 2021.

**Results:**

Screening of SLR outputs identified 72 relevant real-world study publications featuring dose escalation of vedolizumab maintenance therapy. After qualitative review, ten eligible studies (9 articles, 1 abstract) were identified as reporting clinical response and/or clinical remission rates following escalation of intravenous vedolizumab 300 mg Q8W maintenance dosing to every 4 weeks (Q4W) maintenance dosing in adult patients with UC/CD (≥10 patients per study). Overall, 196/395 (49.6%) patients with IBD had a response within 54 weeks of vedolizumab maintenance dose escalation. Although definitions for clinical response/remission varied across the 10 studies, clinical response rates after escalated vedolizumab Q8W maintenance dosing ranged from 40.0% to 73.3% (9 studies) and from 30.0% to 55.8% for remission (4 studies) over a range of 8 to <58 weeks’ follow-up.

**Conclusions:**

This synthesis of real-world effectiveness data in vedolizumab-treated patients with IBD indicates that approximately half were able to achieve or recapture clinical response after escalating vedolizumab maintenance dosing.

## Graphical Abstract

**Graphical Abstract:**
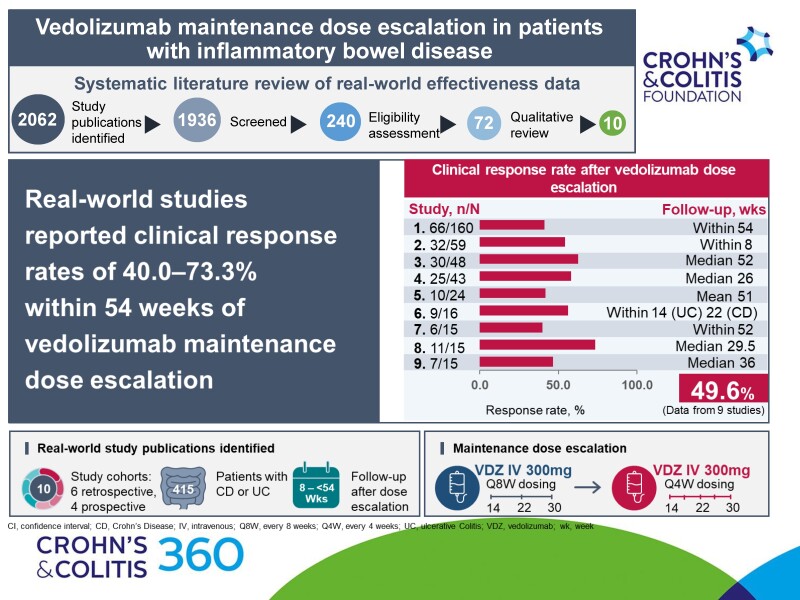

## Introduction

Inflammatory bowel disease (IBD) comprises two conditions, ulcerative colitis (UC) and Crohn’s disease (CD), characterized by chronic inflammation of the gastrointestinal tract.^1^ Therapeutic management seeks to induce and maintain remission of symptoms by immunosuppression, as well as by targeting specific proinflammatory signaling pathways in the gut.^1–5^ Antibody therapies, termed biologics, have been shown to be effective treatments for IBD by acting as inhibitors of intestinal inflammation.^6,7^

A proportion of patients receiving maintenance phase therapy with biologics experience a subsequent loss of response over time, after an initial response.^8^ Average rates of dose escalation with anti-tumor necrosis factor alpha (TNFα) or anti-integrin biologics within 1 year have been estimated at 36% for UC and 30% for CD.^9,10^ The mechanisms underlying secondary loss of response (SLOR) are unclear and are thought to be multifactorial. For anti-TNFα treatments, SLOR has been associated with immunogenicity responses, leading to generation of anti-drug antibodies and increased drug clearance rates.^11,12^ Dose escalation is one pharmacologic approach used to manage SLOR in patients with IBD^8,13^ and is standard practice in patients experiencing loss of response after receiving anti-TNFα therapies such as adalimumab or infliximab, for which success of dose intensification is documented.^9,10,14,15^

Vedolizumab is a gut-selective anti-lymphocyte trafficking agent approved for the treatment of moderate-to-severe UC and CD.^16,17^ This humanized anti-α_4_β_7_ integrin monoclonal antibody selectively antagonizes gastrointestinal integrin receptors, resulting in reduced lymphocyte trafficking into intestinal tissue.^18^ GEMINI clinical trials established the efficacy and safety of 300 mg intravenous (IV) infusion dosing with vedolizumab for the treatment of adult patients with moderate to severely active UC and CD using either every 4 weeks (Q4W) or every 8 weeks (Q8W).^19–21^

Review of data from the clinical real-world experience of vedolizumab dose escalation, collected in uncontrolled conditions, is important to fill data gaps for patient experiences normally excluded from clinical trials. Such data are also critical to understanding the effectiveness and safety of dose escalation achieved in clinical practice, including dose escalation specifically for SLOR.

The objectives of this study were thus: (1) to conduct a comprehensive systematic literature review to identify published real-world studies reporting clinical effectiveness and safety outcomes associated with intensification of standard vedolizumab maintenance phase dosing Q8W to more frequent dosing focusing on the Q4W regimen in adult patients with UC or CD; and (2) to report the rates of clinical response and remission achieved in patients with IBD following dose escalation of vedolizumab maintenance treatment in real-world studies.

## Materials and Methods

### Study Selection

A systematic literature search of the MEDLINE and EMBASE databases was conducted on August 25, 2021. English-language publications (including EMBASE-indexed conference abstracts) from January 1, 2014 to August, 25, 2021 were identified using a pre-defined search strategy (Table S1) to detect studies reporting clinical effectiveness outcomes associated with dose escalation of vedolizumab maintenance Q8W dosing in adult patients with UC or CD. The EMBASE search included abstracts from the following recent congresses of potential relevance to the topic: American College of Gastroenterology 2019 and 2020; Academy of Managed Care Pharmacy (AMCP) 2019; Crohn’s and Colitis Congress 2020; Digestive Disease Week (DDW) 2019 and 2020; International Society for Pharmacoeconomics and Outcomes Research (ISPOR) 2018 and 2020; ISPOR Europe 2019 and 2020; ISPOR Asia Pacific 2020; United European Gastroenterology Week (UEGW) 2019. Systematic reviews, meta-analyses, and abstracts from the following congresses: European Colitis and Crohn’s Organisation 2019, 2020, and 2021, DDW 2021; ISPOR 2019 and 2021, and UEGW 2020 and 2021 were also hand-searched to identify any additional studies. Study publications were reviewed independently by two researchers to determine their suitability for inclusion according to a set of population, intervention, comparator, outcome, and study design selection criteria (PICOS) (Table S2). In the event of a lack of consensus between the reviewers, the decision on study selection was made by a third independent reviewer. Publications were qualitatively assessed for eligibility in a 2-step process. The first stage of qualitative review identified real-world studies of patients aged ≥18 years treated with vedolizumab 300 mg IV for UC or CD who underwent dose escalation. The second stage of qualitative review applied more stringent study inclusion criteria in relation to study sample size and the conditions of dose escalation and follow-up.

### Data Extraction and Outcome Measures

Two researchers extracted data from the study publications selected for inclusion according to a list of pre-specified variables including type of publication, number of patients, study duration, definition of outcome, and proportions (numerator and denominator) of patients achieving two effectiveness outcomes: rate of clinical response and rate of clinical remission after dose escalation. Following qualitative assessment (see PICOS criteria listed in Table S2), studies with a sufficient sample size (≥10 patients) and numerical data reporting clinical response and clinical remission rates after dose escalation to vedolizumab Q4W maintenance dosing following initial Q8W dosing were selected for data extraction.

### Study Quality Assessment

The quality of the full-text study publications reviewed was assessed against key criteria in accordance with US Food and Drug Administration guidance for evaluation of real-world evidence and applicable Wang criteria.^22,23^

### Statistical Analyses

The proportions of patients achieving response and remission within 52 weeks of vedolizumab dose escalation in eligible real-world studies were presented in summary tables.

## Results

### Study Characteristics

**Figure 1.**
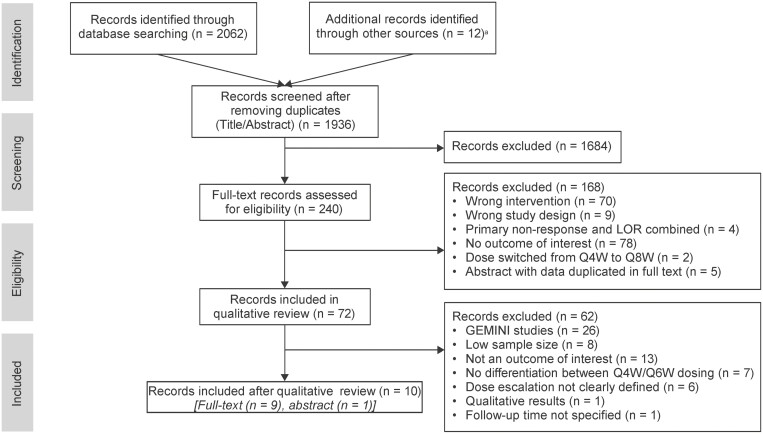
PRISMA flow diagram. ^a^Included from references of systematic reviews and hand-searched congress abstracts. Abbreviations: LOR, loss of response; Q4W, every 4 weeks; Q6W, every 6 weeks; Q8W, every 8 weeks.

A total of 2062 publications were identified in the literature search (1936 from databases and 12 from additional sources, including publications identified from handsearching of systematic reviews and congress abstracts). After initial screening for relevance and removal of duplicates, 240 publications were further assessed for eligibility and 72 were selected for inclusion in the qualitative literature review; reasons for exclusion are summarized in Figure 1. Of 72 publications, 26 reported outcomes from GEMINI open-label studies, and the remaining 46 publications were real-world studies featuring data on vedolizumab dose escalation. Data extraction and narrative synthesis of these 46 real-world studies retrieved from the literature review (combined data in Table S3 and Table 1) indicated a mix of multi- and single-center studies, including retrospective, cohort, or chart review studies, as well as prospective, cross-sectional observational, or registry studies.

**Table 1.** Real-world studies reporting clinical response and/or clinical remission rates after vedolizumab dose escalation: study details and patient characteristics.

Study	Country	Study design	Total study population size, *N*	Patients with SLOR receiving VDZ Q8W, *n/N* (%)	Dose escalation sample size, *n*	Patient characteristics in overall study population (dose-escalated cohort was a sub-population)			
						Age, years, median (IQR)^a^	Disease duration, years, median (IQR)	BL disease activity	Prior anti-TNFα, *n/N* (%)
Amiot et al (2017)^24^	France	Multicenter prospective cohort study (OBSERV-IBD)	272 CD: 161 UC: 111	NR	160^b^ (Q4W) (includes both UC and CD patients)	CD: 35.6 (29.4–46.8) UC: 41.8 (30.2–54.8)	CD: 10.7 (7.3–16.8) UC: 8.4 (4.7–13.2)	CD: BL HBI score: 6.0 (3.0–9.0) UC: BL partial Mayo score: 3.0 (1.0–5.0)	CD: 160/161 (99) UC: 109/111 (98)
Christensen et al (2018)^25^	United States	Single-center prospective cohort study	136 CD: 94 UC: 42	52 (44)	43^c^ (40 Q4W; 3 Q6W) CD: 30 UC: 13	CD: 41 (26–51) UC: 37 (29–45)	CD: 14 (8–24) UC: 9 (5–16)	CD: BL HBI score: <5 (remission): 39/94 (41) 5–7 (mild): 31/94 (33) 8–16 (moderate): 20/94 (21) >16 (severe): 4/94 (4) UC: BL SCCAI score: <3 (remission): 7/42 (17) 3–6 (mild): 22/42 (52) 7–10 (moderate): 9/42 (21) >10 (severe): 4/42 (9)	CD: 90/94 (96) UC: 29/42 (69)
Dreesen et al (2018)^26^	Belgium	Single-center retrospective chart review study	179 CD: 113 UC: 66	NR	16^d^ Q4W CD: 13 UC: 4	CD: 40 (29–52) UC: 41 (30–53)	CD: 14 (6–22) UC: 9 (4–19)	NR	CD: 101/113 (89) UC: 52/66 (79)
Kopylov et al (2019)^27^	Israel	Multicenter retrospective cohort study	193 CD: 133 UC: 60	12/62 (19)	48^e^ (Q4W/Q6W)	CD: 40 (29–57) UC: 36 (30–49)	NR	NR	CD: 127/133 (96) UC: 51/60 (85)
Williet et al (2017)^28^	France	Multicenter prospective study	47 CD: 31 UC: 16	NR	15^f^ (Q4W)	39 (32–46.5)^g^	NR	NR	NR
Dragoni et al (2019)^29^	Italy	Single-center retrospective study	49 CD: 27 UC: 22	15/49 (31)	15^h^ (Q4W)	CD: 44 (30–69) UC: 58 (38–68)	CD: 15 (8–27) UC: 8 (4.5–13.5)	CD: Severe: 25/27 (93) Non-severe 2/27 (7) UC: Severe: 20/22 (91) Non-severe: 2/22 (9)	CD: 21/27 (78) UC: 13/22 (59)
Outtier et al (2021)^30,^^i^	Belgium	Multicenter prospective study	59^j^ CD: 28 UC: 31	59	59 (Q4W)	CD: 25.2 (17.5–30.1) UC: 33.2 (21.5–41.2)	CD: 8.2 (5.4–14.7) UC: 5.9 (3.5–12.5)	CD: BL HBI: 8 (5–13) UC: BL partial Mayo score: 6 (5–6)	CD: 22 (79) UC: 23 (74)
Attauabi et al (2021)^31^	Denmark	Multicenter retrospective cohort study	182 CD: 85 UC: 97	NR	20^k^ CD: 8 UC: 12 (Q4W)	CD: 33 (26–44) UC: 32 (25–44)	10.5 (5.0–19.4) 4.0 (1.9–7.7)	CD: HBI 5 (8–11) UC: SCCAI 6 (4–8)	CD: 85/85 (100) UC: 97/97 (100)
Perry et al (2021)^32^	United States	Single-center retrospective cohort study	90 CD: 0 UC: 90	3/90 (3)	24^l^ (Q4W)	UC: mean 43.3	Mean 12.2	BL Mayo score: mean 6.4 BL partial Mayo score: mean 4.6	20 (83)
Shivashankar et al (2017)^,33,^^m^^,^^n^	United States	Single-center retrospective cohort study	151 CD:120 UC:31	27/151 (18)	15^o^ (Q4W)	Median 40 (range, 19–76)	NR	Mean week 0 HBI score: 8.9	NR

Age in years at first VDZ treatment expressed as median (IQR), unless otherwise stated.

Dose optimization to VDZ 300 mg Q4W for non-response, 82/160 patients; dose optimization for inadequate response, 78/160 patients.

A total of 36/43 (84%) patients were dose escalated for active clinical disease, 5/43 (12%) for glucocorticoid dependence, and 2/43 (5%) for non-healing perianal disease.

Rationale for dose intensification not specified; 1 patient received treatment intensification from Q8W to Q4W after assessment of clinical and endoscopic outcome hence effects of dose escalation assessed in 16 patients not 17.

Dose escalation from Q8W at discretion of treating physician.

Dose escalation from Q8W for loss of response.

Age in dose-escalated patients.

Dose escalation from Q8W for partial loss of response.

Age at diagnosis.

Patients with SLOR on Q8W maintenance (CD: HBI score of >4 with objective signs of inflammation as measured by endoscopy, ultrasound, radiography, CRP >5 mg/L or FCP >250 µg/g assessed at week 14; UC: total Mayo score >6 assessed at week 10) recruited specifically for dose escalation study.

Dose escalation from Q8W based on decision by panel of treating physicians considering SCCAI and HBI scores, objective markers of inflammation (CRP, FCP, MRI, intestinal ultrasound and endoscopy data).

Dose escalation from Q8W for partial response (≥2-point reduction of partial Mayo score) 19/24 (79%); SLOR 3/24 (13%); or no response to Q8W dosing 2/24 (8%).

Main study objective was clinical outcomes in VDZ-treated patients after maintenance phase dose escalation (in all other studies, clinical outcomes after VDZ dose escalation were not the main study objective).

Abstract publication; all other studies were published as full journal articles.

Dose escalation from Q8W for loss of response based on decision of treating physician.

Abbreviations: BL, baseline; CD, Crohn’s disease; HBI, Harvey-Bradshaw Index; IQR, interquartile range; NR, not reported; Q4W, every 4 weeks; Q6W, every 6 weeks; Q8W, every 8 weeks; SCCAI, Simple Clinical Colitis Activity Index; SLOR, secondary loss of response after initial response at vedolizumab induction; TNF, tumor necrosis factor; UC, ulcerative colitis; VDZ, vedolizumab.

In the 46 real-world studies, sample size (for patients with IBD receiving maintenance phase dose escalation) varied from 1 to 161, with follow-up ranging from 12 to 104 weeks. Selection of real-world studies excluded a further 36 study publications (Figure 1): 8 had a low sample size in the dose-escalated cohort (≤10 patients), 13 did not report the outcome of clinical response and clinical remission, 7 combined Q4W and every 6 weeks (Q6W) dosing cohorts, 6 failed to specify the reason for dose escalation, and 2 studies had insufficient data on the outcomes of clinical response and clinical remission (Figure 1). A total of 10 real-world study publications (9 full-text articles and 1 congress abstract) were selected for inclusion^24–33^ that were published from 2017 to 2021. The quality of the full-text study publications was assessed using FDA checklist, which included evaluation of potential sources of bias (Table S4).

### Clinical Response and Remission Rates After Vedolizumab Q8W to Q4W Dose Escalation From 10 Real-World Studies

Of the 10 included studies (5 single-center, 5 multicenter)^24–33^ reporting clinical response and/or remission rates associated with dose escalation of standard vedolizumab 300 mg Q8W maintenance phase treatment (Table 1), most were retrospective, cohort, or chart review studies,^26,27,29,31–33^ and 4 were prospective cohort studies.^24,25,28,30^ Studies were conducted in centers in the United States, Belgium, France, Denmark, Israel, and Italy. Demographics and clinical characteristics of patients with IBD from the 10 studies are provided in Table 1; these data mostly represent the overall study population from which dose-escalated patient cohorts were derived. One study compared the baseline characteristics of patients achieving clinical remission on Q8W vedolizumab maintenance dosing with dose-escalated patients.^32^

For most studies (8/10), response and remission outcomes in dose-escalated patients were not assessed as the primary study objective; in the study recruiting patients with SLOR, the proportion of patients achieving recaptured response after Q4W dose escalation was assessed as a secondary endpoint.^30^ One study^33^ reported clinical effectiveness and safety outcomes exclusively for the dose-escalated cohort and assessed recapture of clinical response in patients with IBD after vedolizumab dose escalation as its primary objective. Another assessed response and remission rates separately in patients receiving standard Q8W dosing and Q4W escalated dosing as the primary study outcome (although dose escalation was not exclusive for SLOR).^32^ Patient eligibility criteria and the definitions for clinical response and remission, SLOR, and the conditions leading to intensification of vedolizumab maintenance dosing varied across the 10 real-world studies (Table 2). Sample size for the cohort of dose-escalated patients in each study ranged from 15 to 160 (Table 1), and total length of follow-up after vedolizumab dose escalation ranged from 8 to <58 weeks (Table 3).

**Table 2.** Real-world studies on vedolizumab dose escalation included after qualitative analysis of SLR outputs: dosing information and clinical response and remission definitions.

Study	Vedolizumab dosing	Clinical response definitions	Clinical remission definitions
Amiot et al (2017)^24^	Induction: 300 mg IV infusion at weeks 0, 2, and 6 Maintenance: 300 mg IV infusion Q8W from week 14 through week 54 Maintenance dose escalation: 300 mg IV infusion Q4W for patients without sufficient response at discretion of investigator	CD: ≥3-point reduction in baseline HBI score UC: ≥3-point reduction in baseline partial Mayo score^a^ and a decrease of ≥30%, with a ≥1-point reduction in baseline rectal bleeding subscale or absolute rectal bleeding score of 0 or 1	CD: HBI score of ≤4 points UC: partial Mayo score^a^ of <3 points, with a combined stool frequency and rectal bleeding subscore of ≤1 point
Christensen et al (2019)^25^	Induction: 300 mg IV infusion at weeks 0, 2, and 6 Maintenance: 300 mg IV infusion Q8W from week 14 through week 52 Maintenance dose escalation: 300 mg IV infusion Q4W or Q6W at discretion of treating physician in patients with clinically active UC/CD, glucocorticoid dependence, or moderate to severe endoscopic disease activity	CD: ≥3-point reduction in HBI score UC: ≥3-point reduction in SCCAI	CD: HBI score of ≤4 points UC: SCCAI score of ≤2 points
Dreesen et al (2018)^26,^^b^	Induction: 300 mg IV infusion at weeks 0, 2, and 6 Induction dose escalation: additional 300 mg IV dose at week 10 for patients with CD with no response at week 6 Maintenance: 300 mg IV infusion Q8W from week 14 through week 30 Maintenance dose escalation: 300 mg IV infusion Q4W, rationale not specified	CD: marked decrease or disappearance of symptoms (Physician Global Assessment) by week 22 UC: marked decrease or disappearance of symptoms (Physician Global Assessment) by week 14	N/A
Kopylov et al (2019)^27^	Induction: 300 mg IV infusion at weeks 0, 2, and 6 Induction dose escalation: additional 300 mg IV dose at week 10 Maintenance: 300 mg IV infusion Q8W from week 14 through week 52 Maintenance dose escalation: 300 mg IV infusion Q4W or Q6W at discretion of treating physician	CD: ≥3-point reduction in HBI score UC: ≥3-point reduction in SCCAI score or ≥2-point improvement in partial Mayo score SLOR: clinical exacerbation following initial clinical response achieved by week 14 (induction); also need for surgery, initiation of corticosteroids, or immunomodulators	CD: HBI score of ≤4 points, CDAI score of <150 points UC: SCCAI score of <2 points, partial Mayo score of ≤2 points
Williet et al (2017)^28^	Induction: 300 mg IV infusion at weeks 0, 2, and 6 Induction dose escalation: additional 300 mg IV dose at week 10 if no response Maintenance: 300 mg IV infusion Q8W from week 14 through week 52 Maintenance dose escalation: 300 mg IV infusion Q4W for loss of response	CD: lack of clinical response was defined as CDAI score of >220 points, with a fecal calprotectin >250 µg/g of stool UC: lack of clinical response was defined as a total Mayo score of >4 points with an endoscopy score of >1 point	N/A
Dragoni et al (2019)^29^	Induction: 300 mg IV infusion at weeks 0, 2, and 6 Induction dose escalation: additional 300 mg IV dose at week 10 for patients with CD with no response at week 6 Maintenance: 300 mg IV infusion Q8W from week 14 through week 52 Maintenance dose escalation: 300 mg IV infusion Q4W for partial loss of response	CD: HBI score of <7, or a ≥3-point reduction in HBI score UC: partial Mayo score of <4, or a reduction in activity of ≥30%	CD: HBI score of <4 UC: partial Mayo score of 0–1
Outtier et al (2021)^,30,^^c^	Induction: 300 mg IV infusion at weeks 0, 2, and 6 (not stated if additional 300 mg IV dose at week 10 was administered) Maintenance: 300 mg IV infusion Q8W Maintenance dose escalation: 300 mg IV infusion Q4W for SLOR	CD: ≥3-point reduction in HBI score UC: ≥2-point reduction in partial Mayo score SLOR: CD: HBI score of >4 with objective signs of inflammation as measured by endoscopy, ultrasound, radiography, CRP >5 mg/L or FCP >250 µg/g assessed at week 14 UC: total Mayo score >6 assessed at week 10	N/A
Attauabi et al (2021)^31^	Induction: 300 mg IV infusion at weeks 0, 2, and 6 Induction dose escalation: additional 300 mg IV dose at week 10 for patients with partial response^d^ or no response at week 6 Maintenance: 300 mg IV infusion Q8W from week 14 Maintenance dose escalation: 300 mg IV infusion Q7W, Q6W, Q5W, or Q4W for partial response based on decision of physician panel, considering SCCAI and HBI scores, CRP, FCP, MRI, intestinal ultrasound and endoscopy data	CD: ≥3-point reduction in baseline HBI score UC: ≥2-point reduction in baseline SCCAI score	CD: HBI score of ≤4 UC: SCCAI score of ≤2
Perry et al (2021)^32^	Induction: 300 mg IV infusion at weeks 0, 2, and 6 Maintenance: 300 mg IV infusion Q8W from week 14 Maintenance dose escalation: 300 mg IV infusion Q4W for partial response, SLOR or no response assessed case-by-case by treating physicians considering disease flares and extra-intestinal symptoms	UC: ≥2-point reduction in baseline partial Mayo score SLOR: relapse after initial remission (partial Mayo score <2)	UC: partial Mayo score of 0 or 1 in patients not receiving CS
Shivashankar et al (2017)^33^	Induction: 300 mg IV infusion at weeks 0, 2, and 6 Maintenance: 300 mg IV infusion Q8W from week 14 Maintenance dose escalation: 300 mg IV infusion Q4W or Q6W for loss of response based on decision of treating physician	A ≥3-point reduction in baseline HBI score^e^ Recaptured response determined by treating gastroenterologist’s review of clinical documentation	N/A

Partial Mayo score (0–9) was the Mayo score minus the endoscopic subscore.

Biologic response was evaluated in a subset of patients with CD with elevated CRP (>5 mg/L) at week 0, defined as a ≥50% decrease in baseline CRP or CRP normalization to ≤5 mg/L(biologic remission). Biologic response and remission were assessed at study weeks 6 and 22. Mucosal healing, as defined for UC, was: Mayo endoscopic subscore of 0 or 1 based on week 14 sigmoidoscopy; for CD this was defined as complete absence of ulcerations based on ileocolonoscopy around study week 22.

Biological response evaluated in patients with CD or UC defined as CRP ≤5 mg/L or a decrease in CRP of >50% in patients with baseline CRP >5 mg/L.

Any numerical reduction in baseline HBI or SCCAI score that was not classified as a clinical response.

No specific definition for clinical response in patients with UC could be located in the source abstract for this study.

Abbreviations: CD, Crohn’s disease; CDAI, Crohn’s Disease Activity Index; CRP, C-reactive protein; CS, corticosteroids; FCP, fecal calprotectin; HBI, Harvey–Bradshaw Index; IV, intravenous; MRI, magnetic resonance imaging; N/A, not applicable; Q4W, every 4 weeks; Q6W, every 6 weeks; Q8W, every 8 weeks; SCCAI, Simple Clinical Colitis Activity Index; SLOR, secondary loss of response; UC, ulcerative colitis.

**Table 3.** Rates of clinical response and/or remission after vedolizumab dose escalation reported in included real-world studies from SLR.

Study	Dose escalation sample size, *n*	Follow-up time after dose escalation, weeks	Patients with clinical response after dose escalation, *n* (%)	Patients with clinical remission after dose escalation, *n* (%)	Safety outcomes reported
Amiot et al (2017)^24^	160^a^	<54	66 (41.3)	48 (30.0)	Yes, not reported separately for dose-escalated cohort
Christensen et al (2018)^25^	43^b^	26 (median)	25 (58.1)	24 (55.8)	Yes, not reported separately for dose-escalated cohort
Dreesen et al (2018)^26,^^c^	16 (CD: 13) (UC: 4)	<14 (UC) or <22 (CD)	9 (56.3)	NR	Yes, not reported separately for dose-escalated cohort
Kopylov et al (2019)^27^	48^d^ (CD: 31) (UC: 17)	52	30 (62.5)	NR	Yes, not reported separately for dose-escalated cohort
Williet et al (2017)^28^	15	36 (median)	7 (46.7)	NR	NR
Dragoni et al (2019)^29,^^e^	15	<52	6 (40.0)	NR	Yes, not reported separately for dose-escalated cohort
Outtier et al (2021)^30^	59	8	32 (54.2)	NR	NR
Attauabi et al (2021)^31^	20	<58^f^	NR	11 (55.0)	Yes, not reported separately for dose-escalated cohort
Perry et al (2021)^32^	24	51 (mean)	10 (41.7)	10 (41.7)	Yes, not reported separately for dose-escalated cohort
Shivashanar et al (2017)^33,^^g^^,^^h^	15^i^	29.5 (mean)	11/15 (73.3)	NR	2 AEs in dose-escalated cohort (hives and pruritus)

Included patients with partial response and secondary loss of response.

Of 43 patients who were dose escalated, 40 received vedolizumab Q4W maintenance therapy and 3 received every 6 weeks.

Did not specify how long after dose escalation response/remission this was observed (only the time period for the whole study was described). Effect of dose escalation assessed in 16 out of 17 patients dose escalated.

Study reports that 17 patients with UC were escalated to Q4W dosing; as dose escalation regimen was not specified for 31 patients with CD, it was assumed that Q4W regimen was also used.

Study mentioned “clinical benefit,” which was confirmed to mean “clinical response” upon further correspondence with the primary author.

Median time to dose escalation was 46 weeks (UC) and 27 weeks (CD).

Main study objective was to study clinical outcomes in vedolizumab-treated patients after dose escalation.

Abstract publication (all other studies were published as full journal articles).

In total, 27 patients received dose escalation of vedolizumab maintenance therapy in this study. Data from 15 patients who were dose escalated to the Q4W regimen were included.

Abbreviations: AE, adverse event; CD, Crohn’s disease; NR, not reported; Q4W, every 4 weeks; UC, ulcerative colitis.

All 10 studies reported standard vedolizumab treatment organized into induction phase therapy (vedolizumab 300 mg IV infusion at weeks 0, 2, and 6) followed by maintenance phase therapy (vedolizumab 300 mg IV infusion Q8W from week 14) (Table 2). Five studies reported an additional vedolizumab 300 mg IV dose at week 10 for patients with no response at week 6 (Table 2). Descriptions of dose escalation varied between studies: most described intensification of vedolizumab 300 mg IV maintenance phase dosing to Q4W, 4 studies listed other dosing frequencies in their methodology, commonly Q6W (Table 2). As expected for real-world studies, patient eligibility for dose escalation during maintenance therapy varied across the 10 studies, typically pertaining to SLOR, partial response or non-response, and often at the discretion of the treating physician. Individual study eligibility criteria for dose escalation, and any definitions provided for loss of response or recaptured clinical response, are detailed in Table 2. Four studies explicitly reported clinical response data following Q8W to Q4W dose escalation in patients with SLOR.^28–30,33^

There was some degree of commonality in the definitions of clinical response and remission used by individual real-world studies (Table 2). For CD, these definitions frequently included Harvey-Bradshaw Index (HBI) scores (6 studies).^24,25,27,29–31^ A reduction in HBI score of ≥3 points for clinical response and HBI score of ≤4 with or without a Crohn’s Disease Activity Index (CDAI) score <150 for clinical remission. One study defined clinical response as a CDAI score of <220 and included the inflammatory biomarker fecal calprotectin at <250 µg/g of stool.^28^ For UC, definitions of clinical response commonly included a ~25%–30% reduction in baseline partial Mayo score^24,25,27–30,32^ and/or improvement in Simple Clinical Colitis Activity Index (SCCAI).^25,27,31^ Accompanying decreases in rectal bleeding subscore or absolute rectal bleeding subscore of ≤1 were used in 1 study,^24^ and 1 study^28^ also included an endoscopy score of <1. Definitions of remission in UC commonly included partial Mayo scores of 0–1 or <3^24,27,29,32^ and/or SCCAI score of <2 or <3.^25,27,31^ One study added a combined stool frequency and rectal bleeding subscore of ≤1 to a partial Mayo score of <3 to define remission.^28^ Although 9 of the 10 studies used objective outcome measures, one study used the subjective Physician Global Assessment of decrease in symptoms to define clinical response in both UC and CD.^26^

For the included studies, reported rates of clinical response (9 studies) after escalation of vedolizumab 300 mg Q8W maintenance dosing ranged from 40% to 73.3% over a range of 8 to <54 weeks’ follow-up (Table 3). In total, 196 of 395 (49.6%) patients from 9 study cohorts had a clinical response after dose escalation during vedolizumab Q8W maintenance phase treatment. The 4 studies reporting response rates after vedolizumab dose escalation specifically to Q4W dosing for SLOR,^28–30,33^ reported recaptured response rates ranging from 40.0% to 73.3% over a range of 8 to <52 weeks’ follow-up.

Of the 4 studies reporting on clinical remission after vedolizumab dose escalation, rates were from 30.0% to 55.8% over a range of 26 to <58 weeks’ follow-up. Overall, 93/247 (37.7%) of patients from the 4 study cohorts achieved clinical remission (Table 3).

### Safety Outcomes

Overall, 8 of 10 studies included following qualitative assessment reported patient safety outcomes (Table 3). Of these, 7 of 8 did not evaluate safety outcomes separately for patients in the vedolizumab maintenance dose escalation cohort. Two adverse events (AEs; hives and pruritus) were reported from a cohort of 15 patients with IBD who were escalated to Q4W vedolizumab maintenance dosing, per a congress abstract report of a single-center retrospective cohort study.^33^ Two studies (evaluated in the first stage qualitative review of real-world dose-escalation studies) reported safety information from patients undergoing vedolizumab dose escalation (Table S4). One multicenter retrospective cohort study quantified rates of infectious and non-infectious AEs in 1,087 vedolizumab-treated patients with IBD. Although the total number of patients undergoing dose escalation was not specified, the study noted 2 patients who developed AEs (sinusitis and arthralgia) after dose escalation to Q4W dosing.^34^ An abstract report of a retrospective chart review, including 62 patients with IBD treated with vedolizumab for ≥52 weeks, noted 3 of 14 dose-escalated patients with AEs, although the type was not specified.^35^

## Discussion

This SLR identified published real-world clinical effectiveness studies reporting rates of clinical response and remission in vedolizumab-treated patients with IBD after dose escalation in the maintenance phase of treatment for SLOR or non-response. In general, the literature review found that independent studies using a variety of data sources (patient registries and chart reviews) reported similar results for clinical response and remission rates achieved after dose escalation of vedolizumab Q8W maintenance treatment in patients with IBD. Patients with SLOR on vedolizumab Q8W maintenance dosing reported from real-world study publications ranged from 13% to 44%. From the 10 real-world studies included after qualitative assessment of SLR outputs, ~40%–70% of patients with IBD achieved a clinical response (reported in 9 studies) and ~30%–55% achieved clinical remission (reported in 4 studies) following Q8W vedolizumab maintenance phase dose escalation. Recaptured response rates after escalation to Q4W dosing for SLOR (specifically reported in 4 studies) also fell within the range of ~40%–70%.^28–30,33^

These results are in line with a previous systematic literature review and meta-analysis of literature published up to December 2017, featuring 8 real-world and 2 interventional cohort studies of dose intensification for SLOR during Q8W maintenance to Q4W, Q6W, or unspecified dosing regimens, which reported a recaptured response rate of 53.8% (95% CI: 21.8%–82.9%; I^2^ = 77%).^36^

SLOR in a proportion of patients with IBD is a feature common to treatment with biologics, with an incidence of 23%–46% at 1 year in patients receiving anti-TNFα treatment.^37^ The clinical benefit of adalimumab and infliximab dose escalation following SLOR was established in prospective studies,^38,39^ and rates of response following dose escalation in real-world settings are in line with those achieved with vedolizumab.^9,10,14,15^ Loss of response to biologics over time has been linked to immunogenicity and increased clearance by anti-drug antibodies, as well as to other factors related to patient and disease characteristics, including body size, degree of systemic inflammation, and concomitant use of immunomodulators.^37,40,41^ Therapeutic monitoring to identify patients with SLOR who have low drug trough concentrations has been used to demonstrate an association between clinical benefit and a rise in trough concentrations following dose escalation of anti-TNFα treatment in these patients.^8,38,42,43^ Dose escalation of anti-TNFα treatments may also be useful in patients with low titer anti-drug antibodies by facilitating adequate trough concentrations to allow recapture of clinical response.^8,44^

A correlation between vedolizumab exposure and response was similarly reported in 3 real-world studies included in the SLR, where lower vedolizumab serum trough concentrations were associated with a need for dose escalation within 6 months of treatment.^26,28,30^ In a retrospective cohort study of 179 patients with IBD, those with low vedolizumab trough concentrations from week 2 at induction to week 30 during maintenance therapy had a lower probability of achieving effectiveness end points, including clinical, biologic and endoscopic responses (mucosal healing; absence of ulcers/Mayo endoscopic subscore of ≤1) (Table 2). In patients who were escalated from Q8W to Q4W maintenance dosing, vedolizumab trough concentrations doubled from 6.8 µg/mL (95% CI: 5.7–8.3) to 13.4 µg/mL (95% CI: 12.1–19.4; *n* = 13; *P* = 0.0002). Clinical and endoscopic responses were achieved in 56% and 31% of these patients, respectively.^26^ A recent open-label, prospective study reported significant increases in vedolizumab trough concentrations measured after Q8W to Q4W dose escalation for SLOR (median [interquartile range]) from 8.7 (5.1–12.7) µg/mL at baseline to 19.1 (12.4–22.4; *n* = 56) µg/mL and 23.1 (16.7–28.4; *n* = 53) µg/mL at 4 and 8 weeks after dose escalation, respectively (*P* < 0.0001). These corresponded to rates of 49% and 54% for recaptured clinical response and 27% and 37% for recaptured biological response (decrease in C-reactive protein levels of >50% or to ≤5 mg/L) at the same 4- and 8-week time points after dose escalation, respectively.^30^ It has been suggested that increasing vedolizumab bioavailability may be necessary for maintaining therapeutic efficacy in some patients.^45^ Comparison of the baseline characteristics of dose-escalated with non-dose escalated patients in a real-world setting found more severe disease and prior anti-TNFα treatment predicted the requirement for dose escalation.^32^

Positive effects of Q4W dose escalation on endoscopic outcomes, including mucosal healing, were reported in several real-world studies identified in the literature review (Table 2 and Table S3)^24,26,46–50^ including an increased likelihood of achieving mucosal healing (67%) compared with Q8W dosing (39%).^49^ The study in which higher vedolizumab trough concentrations were significantly associated with endoscopic response also reported mucosal healing in 5/16 patients (31%) following Q4W dose escalation.^26^

No new safety signals in relation to vedolizumab dose intensification could be discerned from the real-world studies identified in this literature review. For the safety end points examined in GEMINI 1 and 2 at 52 weeks, there were no important differences noted between the vedolizumab Q8W or Q4W treatment groups,^19,20^ and also no notable differences in safety end points associated with vedolizumab dose escalation to Q4W in the single-arm, phase 3, open-label, long-term extension study GEMINI LTS.^51,52^ Efficacy outcomes were studied in a subgroup of patients in GEMINI LTS (termed the VDZ/Q8W to Q4W population) who withdrew early during the Q8W maintenance phase of the parent GEMINI 1 or 2 studies because of disease worsening, sustained non-response after week 14 (defined as failure to achieve a clinical response, or at the discretion of the study investigator), or the need for rescue medication beyond week 14.^51–53^ These patients had a response to vedolizumab at induction. For patients with UC in GEMINI 1, clinical response was defined as a reduction in Mayo score of ≥3 points and a decrease of ≥30% from the baseline score, accompanied by a ≥1-point decrease in rectal bleeding subscore, or absolute rectal bleeding subscore of 0 or 1. For patients with CD in GEMINI 2, clinical response was defined as a ≥100-point decrease in the CDAI score.^19,20^ Rates of recaptured response within 52 weeks studied in the VDZ/Q8W to Q4W subgroup analysis of the GEMINI LTS clinical trial were within the lower range of those reported for patients after escalation of vedolizumab Q8W maintenance dosing in real-world studies. For the 32 patients with UC, 41% achieved a response (defined as a reduction in baseline partial Mayo score of ≥2 points and ≥25%, accompanied by a ≥1-point decrease in rectal bleeding subscore or absolute rectal bleeding subscore of ≤1) and 28% achieved remission after 52 weeks (defined as a partial Mayo score of ≤2 with no individual subscore of >1).^51^ For the 57 patients with CD, 47% achieved a response (≥3-point decrease from baseline in HBI score) and 32% achieved remission (HBI score of ≤4) within 52 weeks of escalated dosing.^52^

### Limitations

There were several limitations related to the quality of the evidence that can be retrieved from real-world studies as compared with clinical trials. For most of the real-world studies identified, rates of clinical response or remission achieved after dose escalation were not the primary study objectives and were often discerned due to subgroup analysis. Patient study eligibility criteria, baseline characteristics, and the definitions applied for clinical response and remission, SLOR, and the conditions leading to intensification of vedolizumab maintenance dosing were variable across studies, as were vedolizumab dosing schedules, with some studies including an additional vedolizumab dose at week 10 for patients with inadequate responses at induction. In addition, control groups were not included, and covariates of dose escalation and the characteristics of secondary responders were, in most cases, not assessed. There were also variable reasons for dose escalation reported in the real-world studies included from SLR; patients were usually dose escalated either because of presumed loss of response to Q8W maintenance dosing, non-response to induction, or for other reasons. Some studies did not separate results according to whether patients were dose escalated for non-response at induction or during maintenance dosing, and the decision to escalate dose was often at the physicians’ discretion, leading to uncertainty about the timing of dose escalation. For evaluations of safety in these studies, prevalence and type of AEs were usually not reported separately for patients in the dose-escalated cohort. Even following qualitative assessment designed to improve data quality and identify studies with unifying features for comparison, the high level of heterogeneity observed across the 10 individual real-world studies identified from SLR outputs prevented any meaningful synthesis of pooled clinical effectiveness data by meta-analysis.

In addition, real-world studies are considered more prone to bias compared with clinical studies, which are guided by a strict protocol. Nevertheless, the quality assessment (Table S4) and analysis of study methodology found some steps were taken to reduce bias in some real-world studies, for example, by using pre-defined objective measures to define patient eligibility for dose escalation,^30^ centralized review of patient data, and collective clinical decisions on dose escalation.^31^ Also, the selection of studies for review was conducted according to pre-defined criteria and carried out independently by two researchers with disagreements adjudicated by a third reviewer. Conversely, strict patient selection criteria for enrollment into clinical trials can serve to reduce heterogeneity of the study population, limiting generalization of trial results to the non-selected patient populations with IBD encountered in real-world clinical practice.^54,55^ Of note, studies identified in the SLR reported consistent outcomes for vedolizumab dose escalation in patients across several different regions (North America, Europe, and Middle East) at different time points relative to vedolizumab becoming licensed for use, and from different data sources (e.g., patient registries and chart reviews).

## Conclusion

This SLR identified published real-world data on the clinical effectiveness of dose escalation of vedolizumab 300 mg IV Q8W maintenance treatment to achieve or recapture response. In 10 eligible studies reporting on these endpoints, identified from qualitative assessment of SLR outputs, approximately half (49.6%) of the patients with IBD had a clinical response within 54 weeks of escalating vedolizumab Q8W maintenance dosing. Clinical effectiveness data reviewed from current real-world clinical practice indicate that some patients can achieve or recapture clinical response after dose escalation of vedolizumab maintenance treatment.

## Supplementary Material

otac020_suppl_Supplementary_Data

## Data Sharing Statement

The datasets, including the redacted study protocol, redacted statistical analysis plan, and individual participants’ data supporting the results reported in this article, will be made available within three months from initial request, to researchers who provide a methodologically sound proposal. The data will be provided after its de-identification, in compliance with applicable privacy laws, data protection and requirements for consent and anonymization.
