# Supplementary material for: Real-World Effectiveness of Vedolizumab Dose Escalation in Patients With Inflammatory Bowel Disease: A Systematic Literature Review
Source: Crohns Colitis 360. 2022 Jul 8;4(3):otac020. doi: 10.1093/crocol/otac020 (PMC9802433; doi:10.1093/crocol/otac020)
Supplement: otac020_suppl_Supplementary_Data [file otac020_suppl_supplementary_data.docx]

Supplementary Data

**Supplementary Table 1. Database Search Terms**

| **Topic** | **Set** | **Terms** |
| --- | --- | --- |
| IBD population search | S1 | (EMB.EXACT.EXPLODE("inflammatory bowel disease")) OR (EMB.EXACT("ulcerative colitis")) OR (EMB.EXACT("Crohn disease")) OR (MESH.EXACT.EXPLODE("Inflammatory Bowel Diseases")) OR (MESH.EXACT("Colitis, Ulcerative")) OR (MESH.EXACT("Crohn Disease")) OR (TI,AB("crohns disease" OR "crohn disease" OR "crohn's disease" OR "crohns disorder" OR "crohn disorder" OR "crohn's disorder" OR "ulcerative colitis" OR IBD OR "inflammatory bowel disease")) |
| Drug search | S2 | EMB.EXACT("vedolizumab") OR ti,ab(vedolizumab) OR vedolizumab |
| Real-world evidence search | S3 | TI,AB("epidemiologic studies") OR TI,AB("case control studies") OR TI,AB("family study") OR TI,AB("longitudinal study") OR TI,AB("retrospective study") OR TI,AB("prospective study") OR TI,AB("cohort analysis") OR TI,AB("cohort study") OR TI,AB("cohort studies") OR TI,AB("case control study") OR TI,AB("follow up") OR TI,AB("observational study") OR TI,AB("observational studies") OR TI,AB("cross sectional study") OR TI,AB("cross sectional studies") OR TI,AB(cohort NEAR/5 (study OR studies)) OR TI,AB(“case control” NEAR/5 (study OR studies)) OR TI,AB(“follow up” NEAR/5 (study OR studies)) OR TI,AB(observational NEAR/5 (study OR studies)) OR TI,AB(epidemiologic* NEAR/5 (study OR studies)) OR TI,AB(“cross sectional” NEAR/5 (study OR studies)) OR TI,AB("disease registry" or "disease registries") OR EMB.EXACT(“case control study”) OR EMB.EXACT(“family study”) OR EMB.EXACT(“longitudinal study”) OR EMB.EXACT(“retrospective study”) OR EMB.EXACT(“prospective study”) OR EMB.EXACT(“cohort analysis”) OR EMB.EXACT(“follow up”) OR EMB.EXACT(“observational study”) OR EMB.EXACT(“epidemiology”) OR EMB.EXACT(“cross-sectional study”) OR EMB.EXACT("disease registry") OR MESH.EXACT(“Case-Control Studies”) OR MESH.EXACT(“Longitudinal Studies”) OR MESH.EXACT(“Retrospective Studies”) OR MESH.EXACT(“Prospective Studies”) OR MESH.EXACT(“Cohort Studies”) OR MESH.EXACT(“Follow-Up Studies”) OR MESH(“Observational Studies”) OR MESH.EXACT(“Epidemiologic Methods”) OR MESH.EXACT(“Cross-Sectional Studies”) |
| Additional study design | S4 | ((TI,AB("clinical trial") OR TI,AB("clinical trial phase i") OR TI,AB("clinical trial, phase ii") OR TI,AB("clinical trial, phase iii") OR TI,AB("clinical trial, phase iv") OR TI,AB("prospective study") OR TI,AB(clinical AND (trial or study or studies)) OR EMB.EXACT(“clinical trial”) OR EMB.EXACT(“prospective study”) OR MESH(“Clinical Trials”) OR MESH.EXACT(“Prospective Studies”))) |
|  | S5 | (EMB.EXACT("open study") OR TI,AB("open-label" OR "open label" OR "open trial" OR "open study") OR TI,AB("non-blind" OR "non blind" OR "non-blinded" OR "non blinded") OR TI,AB("un blinded" OR "un-blinded")) |
|  | S6 | (MESH.EXACT.EXPLODE("Non-Randomized Controlled Trials as Topic") OR TI,AB ("single arm" OR “single-arm”) OR TI,AB(“non comparative” OR “non-comparative”) OR TI,AB(uncontrolled OR noncontrolled OR “non-controlled”)) |
| Combined search | S7 | S1 AND S2 |
|  | S8 | S3 OR (S4 AND (S5 OR S6)) |
|  | S9 | S7 AND S8 |
| Include studies after January 1, 2014 | S10 | (S9 AND (pd(>20140101))) |
| Include studies in English language | S11 | (S10 AND (la.exact("English"))) |

Abbreviation: IBD, inflammatory bowel disease.

**Supplementary Table 2.** Population, Intervention, Comparator, Outcomes, and Study Design (PICOS) Selection Criteria

|  | **Inclusion Criteria** | **Exclusion Criteria** |
| --- | --- | --- |
| Population | - Adult patients (aged ≥18 years) treated with VDZ for UC or CD |  |
| Intervention | - VDZ every 4 weeks dosing |  |
| Comparators | - Any or none |  |
| Outcomes | - Effectiveness/response outcomes   - Absolute and/or change from baseline in HBI/CDAI score or other clinical scores utilized for IBD (CD and UC)   - Evidence of mucosal healing   - Physicians Global Assessment   - Time to first hospitalization/surgery   - Absence of rectal bleeding and stool frequency of 1 or 2 in patients with UC   - Absence of abdominal pain and decreased loose stool frequency (≤1) in patients with CD - Time to clinical response/remission (induction) - Durability of clinical response (maintenance) - Patient-reported outcome measures (eg, IBDQ, SF‑36, EQ-5D, treatment satisfaction) - Safety/tolerability   - Nasopharyngitis, gut-specific infections, joint pain, infusion-related reactions |  |
| Study design | - Observational studies (cohorts, cross-sectionals, case-controls, registries)   - Prospective   - Retrospective - Systematic reviews - Clinical trials that follow routine clinical practices (open-label and/or single-arm clinical trials) - Case studies/series (≥10 patients) | - Case studies/series (<10 patients) |
| Publication type | - Journal studies - Conference abstracts |  |
| Language | - English |  |
| Publication date | - January 1, 2014 to August 25, 2021 |  |
| Country | - Global |  |

Abbreviations: CD, Crohn’s disease; CDAI, Crohn’s Disease Activity Index; EQ-5D, EuroQoL 5-Dimensions; HBI, Harvey-Bradshaw Index; IBD, inflammatory bowel disease; IBDQ, Inflammatory Bowel Disease Questionnaire; SF-36, 36-item Short Form Survey; UC, ulcerative colitis; VDZ, vedolizumab.

**Supplementary Table 3.** Summary of Key Findings From Real-World Studies Excluded During Eligibility Screening

| Study | Study Design | Q4W Dosing Sample Size, n | Follow-Up Time | Patients Achieving Clinical Response, n (%) | Patients Achieving Clinical Remission,  n (%) | Other |
| --- | --- | --- | --- | --- | --- | --- |
| Low Sample Size | | | | | | |
| Mankongpaisarnrung et al 2016^1^ | Single-center cohort study | 4 | NR | NR | NR | Minimal to mild clinical improvement noted in 3 patients |
| Paul et al 2016^2^ | Prospective observational study | 10 | ≥28 weeks | 4 (40) | NR |  |
| Kopylov et al 2018^3^ | Retrospective multicenter study | 9 | 30 weeks (median) | 4 (44) | NR |  |
| Ladd et al 2016^4^ | Retrospective review of medical records | 10 | 23 weeks (median) | 10 (100) | NR |  |
| Samaan et al 2020^5^ | Retrospective cohort study | 8 | 24 weeks | 7 (87) | NR | Of 8 responders at week 12 needing subsequent dose escalation, 7 had response at week 24 |
| Shalabi et al 2021^6^ | Retrospective, single-center chart review | 1 | NR | 1 (100%) | NR |  |
| Bamias et al 2021^7^ | Prospective, multicenter cohort study | 5 | 54 | 3 (60%) | NR |  |
| Attauabi et al 2021^8^ | Retrospective, nationwide cohort study | 4 | 52 weeks | 2 (50%) | NR |  |
| Q4W and Q6W Dosing Combined | | | | | | |
| Chaparro et al 2018^9^ | Prospective registry (ENEIDA) study | 57 | 52 weeks (median) | 17 (31) | 16 (29) |  |
| Shmidt et al 2018^10^ | Multicenter cohort study | 33 | 26 weeks (median) | 16 (49) | 6 (18) |  |
| Schmidt et al 2019^11^ | Multicenter cohort study | 53 | 39 weeks (median) | 19 (36) | NR |  |
| Dulai et al 2016^12^ | Multicenter cohort study | CD: 13  (lack of response/ suboptimal response) | 39 weeks (median) | 4 (31) | 1 (8) |  |
|  |  | CD: 8 (LOR) | 39 weeks (median) | 3 (37) | 1 (12) |  |
| Lee et al 2020^13^ | Retrospective chart review | 72 | Range: 12 to 52 | 37 (52) | 31 (42) |  |
| Lee et al 2021^14^ | Retrospective chart review | 34 | >12 | 17 (50) | 15 (44) |  |
| Zingone et al 2021^15^ | Retrospective, single-center chart review | 24 | 52 | 16 (67) | NR |  |
| Outcomes: Trough Levels | | | | | | |
| Tessolini et al 2018^16^ | Single-center cross-sectional study | 25 | NR | NR | NR | Q4W dosing group had higher trough levels than the Q8W dosing group (20.6 μg/mL vs 8.35 μg/mL, respectively; *P* < 0.0001) |
| Plevris et al 2019^17^ | Single-center cross-sectional study | NR | NR | NR | NR | Q4W dosing group had higher trough levels than the Q8W dosing group (16.1 μg/mL vs 10.4 μg/mL, respectively; *P* = 0.02) |
| Al-Bawardy et al 2018^18^ | Single-center cross-sectional study | 32 | NR | NR | NR | Median trough level was higher in the Q4W group compared with the Q6W/Q8W groups (25.5 μg/mL vs 16.8 μg/mL vs 13.9 μg/mL, respectively; *P* = 0.002) |
| Al-Bawardy et al 2019^19^ | Single-center, cross-sectional study | 40 | NR | NR | NR | Median trough level was significantly higher in the Q4W vs Q8W (n = 115) groups (28.50 μg/mL  vs 12.80 μg/mL, respectively) |
| Torres et al 2019^20^ | Prospective cross-sectional study | 3 | NR | NR | NR | Median trough level was higher in the Q4W vs Q8W cohorts (n = 18; 11.9 μg/mL vs 8.0 μg/mL, respectively; *P*= 0.182) |
| Ungaro et al 2019^21^ | Multicenter cross-sectional study | 49 | 38 weeks  (median) | NR | NR | Patients receiving VDZ Q4W had significantly higher median concentrations than those receiving Q8W dosing (15.0 μg/mL [IQR, 8.5–24.1 μg/mL] vs 10.2 μg/mL [IQR, 6.3–15.2], respectively;  *P* = 0.003) |
| Plevris et al 2019^17^ | Prospective cross-sectional study | NR | Median VDZ duration, 1.6 years | NR | NR | Individuals receiving Q4W dosing had higher median levels than those receiving Q8W dosing (16.1 μg/mL vs 10.4 μg/mL, respectively;  *P* = 0.02) |
| Ansari et al 2020^22^ | Retrospective chart review | 36 | NR | NR | NR | Patients receiving Q4W dosing with higher VDZ levels were more likely to have mild disease or remission endoscopically (*P* = 0.01) |
| Outcomes: Safety | | | | | | |
| Ritter et al 2017^23^ | Multicenter cohort study | 14 | 52 weeks | NR | NR | 21% experienced AEs |
| Meserve et al 2019^24^ | Retrospective multicenter study | NR | 43 weeks  (median) | NR | NR | 2 patients developed AEs upon escalation to Q4W dosing, 1 of whom developed severe arthralgia |
| Outcomes: Other | | | | | | |
| Dimopoulos et al 2019^25^ | Retrospective cross-sectional study | NR | NR | NR | NR | Of the 22 patients experiencing extraintestinal manifestations, 50% received Q4W dosing (vs 45.5% receiving Q8W dosing) |
| Guidi et al 2019^26^ | Prospective multicenter cohort study | 53 | 52 | NR | NR | Patients receiving Q4W dosing were more likely to achieve mucosal healing (67%, vs 39% among Q8W dosing) |
| Biemans et al 2020^27^ | Prospective multicenter cohort study | 61 (Q ≤ 6W) | 104 | NR | NR | There was no significant difference in drug survival rate between patients Q8W dosing vs patients who underwent dosing interval shortening (hazard ratio: 1.18; 95% CI: 0.71-1.95) |
| Dose Escalation: Reason Not Specified | | | | | | |
| Amor et al 2019^28^ | Retrospective observational study | 16 | 1.97 years (median) | 7 (47) | NR | AE incidence experienced by patients with dose intensification vs no intensification (36.8% vs 20.7%) |
| Graziano et al 2019^29^ | Retrospective multicenter study | 20 | NR | 13 (65) | NR | Of 20 patients, 13 had a positive response to escalation: 8 symptomatic response, 1 biologic response, and 4 endoscopic response |
| Tessolini et al^30^ | Cross-sectional observational study | 25 | NR | NR | 10 (40) | Patients receiving VDZ Q4W had significantly higher serum VDZ levels than those receiving Q8W regimen (18.9 µg/mL vs 8.4 µg/mL, respectively) |
| Albshesh et al 2021^31^ | Retrospective, multicenter cohort study | 30 | 52 | NR | NR | 30 patients (100%) achieved clinical remission or response by the end of follow-up |
| Kolehmainen et al 2021^32^ | Retrospective, single-center cohort study | 36 | 52 | 9 (25) | 3 (8) | 15 patients (41.7%) with increased dose frequency had no observed change in clinical response. Moderate improvement was observed in 9 (25.0%) patients and significant improvement (remission) in 3 (8.3%) patients. |
| Ungar et al 2021^33^ | Multicenter observational study | 161 | 24 | NR | (34) | 6-months after dose escalation, clinical remission, inflammatory remission, and endoscopic remission was achieved in 34%, 60% and 30% of patients, respectively. |
| Lack of Quantitative Results | | | | | | |
| Sierra et al 2018^34^ | Single-center cohort study | 30 | 24 weeks | NR | NR | Approximately half of the study population responded to dose escalation |
| Follow-Up Time Not Specified | | | | | | |
| Wice et al 2016^35^ | Single-center cohort study | 18  CD: 14  UC: 4 | NR | 9 (64) of patients with CD | NR |  |

Abbreviations: AE, adverse event; CD, Crohn’s disease; IQR, interquartile range; LOR, loss of response; NR, not reported; Q4W, every 4 weeks; Q6W, every 6 weeks; Q8W, every 8 weeks; VDZ, vedolizumab; UC, ulcerative colitis.

**Supplementary Table 4.** Quality Assessment of Included Studies

| Criteria | Assessment | | |
| --- | --- | --- | --- |
| Amiot et al 2017^36^ |  | | |
| Transparency about study design and analysis before execution | - Key stakeholders were involved in the design and implementation of the study to ensure that the study addresses meaningful questions | | |
| Study reproducibility and reporting transparency | - The authors have reported the majority of the applicable parameters listed by Wang, enabling reproducibility by other researchers, including, but not limited to, study design, exposure and follow-up, outcomes, and missing data analyses - The authors have not critically addressed their methodology in the publication | | |
| Data reliability and relevance for the research question | - The data were collected from a large cohort of patients with IBD being treated with VDZ in a real-life setting, making it suitable to answer the research question | | |
| Study design to support effectiveness | - The study design did not address those who dropped out of the study by only reporting outcomes among patients who reached 54 weeks, but it had fairly large sample size at 54 weeks - The study design did not specify an active comparator group to allow for comparison between other treatment options - Confounders for dose escalation were not measured - AEs were reported for all patients but not specific to the dose-escalated cohort | | |
| Christensen et al 2018^37^ |  | | |
| Transparency about study design and analysis before execution | - All the authors were from 2 medical centers; therefore, the study could have benefitted from the addition of additional stakeholders - The study does not seem to be publicly registered | | |
| Study reproducibility and reporting transparency | - The authors have reported the majority of the applicable parameters listed by Wang, enabling reproducibility by other researchers, including, but not limited to, study design, exposure and follow-up, outcomes, and missing data analyses (intent-to-treat analysis) - The authors have not critically addressed their methodology in the publication | | |
| Data reliability and relevance for the research question | - The data were collected via a prospective cohort study from a single center; therefore, the results may not be generalizable to a larger population - The patient population was chosen from patients with IBD being treated with VDZ in a real-life setting, making it suitable to answer the research question | | |
| Study design to support effectiveness | - The study design did not specify an active comparator group to allow for comparison between other treatment options - Confounders for dose escalation were not measured - The study was not designed to address dose escalation as a primary objective; thus, only a small subsection of the total study population received dose escalation | | |
| Dreesen et al 2018^38^ |  | | |
| Transparency about study design and analysis before execution | - The authors did not seem to publicly register this study - All the authors were from the same institution, and the study could have possibly benefitted from external stakeholders | | |
| Study reproducibility and reporting transparency | - The authors did not describe how their exposure and outcome variables were identified in the electronic health record system; the low reporting transparency decreases the study reproducibility | | |
| Data reliability and relevance for the research question | - This study used electronic health records, which may not be sufficient as a stand-alone study to generate real-world evidence in support of dose escalation, but it is relevant as part of the evidence package in conjunction with the other studies using varying data sources that show similar results | | |
| Study design to support effectiveness | - This study assessed endoscopic outcomes in the loss of response group, which is an objective end point - The study was not designed to address dose escalation, with only a small subsection of the population receiving dose escalation - The study design did not specify an active comparator group to allow for comparison between other treatment options - Confounders for dose escalation were not measured | | |
| Kopylov et al 2019^39^ |  | | |
| Transparency about study design and analysis before execution | - The authors were all from all university settings (multiple university settings in the same country). The study could have benefitted from the involvement of key stakeholders from other relevant areas - The authors did not seem to publicly register this study | | |
| Study reproducibility and reporting transparency | - Because dose escalation was not a primary objective, the authors did not achieve high reporting transparency with respect to this objective - The authors have not critically addressed their methodology in the publication | | |
| Data reliability and relevance for the research question | - The data were collected from a pooled cohort across multiple centers, making the data more generalizable, but only within the country - Included patients were patients with IBD treated with VDZ in a real-life setting, thus making it suitable to answer the research question | | |
| Study design to support effectiveness | - Patients who did not reach 52 weeks of follow-up while receiving active treatment were excluded - The study design did not specify an active comparator group to allow for comparison between other treatment options - Confounders for dose escalation were not measured | | |
| Williet et al 2017^40^ |  | | |
| Transparency about study design and analysis before execution | - The authors did not seem to publicly register this study - All the authors were from the same institution, and the study could have possibly benefitted from external stakeholders | | |
| Study reproducibility and reporting transparency | - Because dose escalation was not a primary objective, the authors did not achieve high reporting transparency with respect to this objective | | |
| Data reliability and relevance for the research question | - This study may not be sufficient as a stand-alone study to generate RWE in support of dose escalation, but is relevant as part of the evidence package in conjunction with the other studies using varying data sources that show similar results | | |
| Study design to support effectiveness | - The study was not designed to address dose escalation as a primary objective; thus, only a small subsection of the total study population received dose escalation - The study design did not specify an active comparator group to allow for comparison between other treatment options - Confounders for dose escalation were not measured | | |
| Dragoni et al 2019^41^ |  | | |
| Transparency about study design and analysis before execution | - All the authors involved in this study are from the gastroenterology department in 2 university hospitals; the study could have benefitted from the involvement of key stakeholders from other relevant areas - The authors did not seem to publicly register this study | | |
| Study reproducibility and reporting transparency | - Because dose escalation was not a primary objective, the authors did not achieve high reporting transparency with respect to this objective - The authors have not critically addressed their methodology in the publication - Certain outcomes, such as “clinical benefit,” with respect to the dose-escalated cohort were ambiguous and were clarified upon further contact with the corresponding author | | |
| Data reliability and relevance for the research question | - The data were collected from a cohort of patients with IBD being treated with VDZ in a real-life setting, making it suitable to answer the research question - The patients were enrolled from 1 IBD referral center; therefore, the results may not be generalizable to the larger population | | |
| Study design to support effectiveness | - The study was not designed to address dose escalation as a primary objective; thus, only a small subsection of the total study population received dose escalation - The study design did not specify an active comparator group to allow for comparison between other treatment options - Confounders for dose escalation were not measured - AEs were reported for all patients, but not specific to the dose-escalated cohort | | |
| Outtier et al 2021^42^ |  | | |
| Transparency about study design and analysis before execution | - Authors were from various centers across Belgium who were involved in the designing and implementation of the study to ensure that the study addressed meaningful questions - The authors did not seem to publicly register this study | | |
| Study reproducibility and reporting transparency | - The authors have reported majority of the applicable parameters listed by Wang enabling reproducibility by other researchers including but not limited to study design, exposure and follow-up, outcomes - The authors have critically addressed their methodology in the publication, namely the short time from dose escalation to evaluation of response | | |
| Data reliability and relevance for the research question | - The data were collected from a prospective cohort of IBD patients being treated with VDZ in a real-life setting making it suitable to answer the research question - The data were collected from a pooled cohort across multiple centers making the data more generalizable but only within the country | | |
| Study design to support effectiveness | - The study design treated patients with missing data as non-responders - The study design did not specify an active comparator group to allow for comparison between other treatment options - Confounders for dose escalation were not measured - Clinical response was measured after only 8 weeks of dose escalated treatment | | |
| Attauabi et al 2021^43^ |  | | |
| Transparency about study design and analysis before execution | - All the authors involved in this study are from the gastroenterology department in 2 university hospitals; the study could have benefitted from the involvement of key stakeholders from other relevant areas - The authors did not seem to publicly register this study | | |
| Study reproducibility and reporting transparency | - The authors have reported majority of the applicable parameters listed by Wang enabling reproducibility by other researchers including but not limited to study design, exposure and follow-up, outcomes and missing data analyses | | |
| Data reliability and relevance for the research question | - The data were collected from a cohort of IBD patients being treated with VDZ in a real-life setting making it suitable to answer the research question - The patients were enrolled from 2 IBD university centers in one country, therefore the results may not be generalizable to the larger population | | |
| Study design to support effectiveness | - The study design did not specify an active comparator group to allow for comparison between other treatment options - Confounders for dose escalation were not measured - The study design did not address those who dropped out of the study by only reporting outcomes among patients who reached specified timepoints of follow-up | | |
| Perry et al 2021^44^ | |  |  |
| Transparency about study design and analysis before execution | | - All the authors involved in this study are from the gastroenterology department in 1 university hospital; the study could have benefitted from the involvement of key stakeholders from other relevant areas - The authors did not seem to publicly register this study |  |
| Study reproducibility and reporting transparency | | - The authors have reported majority of the applicable parameters listed by Wang enabling reproducibility by other researchers including but not limited to study design, exposure and follow-up, outcomes - Missing data analyses was not reported |  |
| Data reliability and relevance for the research question | | - The data were collected from a cohort of IBD patients being treated with VDZ in a real-life setting making it suitable to answer the research question - The patients were enrolled from 1 IBD university center in one country, therefore the results may not be generalizable to the larger population - The analysis was focused on patients who had partial response to standard dosing and were dose escalated rather than patients who experienced loss of response to standard dosing. |  |
| Study design to support effectiveness | | - The study design did not specify an active comparator group to allow for comparison between other treatment options - Confounders for dose escalation were not measured - Efficacy could only be measured with partial Mayo score rather than full Mayo score due to small sample size |  |

Abbreviations: AE, adverse event; IBD, inflammatory bowel disease; VDZ, vedolizumab

**Reference List for Supplementary Data**

1. Mankongpaisarnrung C CA, Mattar M. Single-Center Experience: Vedolizumab in Patients with Crohn's Disease and Ulcerative Colitis at Georgetown University Hospital. *Inflamm Bowel Dis*. 2016;22(suppl_1):S32-S32.

2. Paul S, Williet N, Claudez P, et al. Sa1939 serum vedolizumab assay at week 6 predicts sustained clinical remission and lack of recourse to optimisation in IBD. *Gastroenterology*. 2016;150(4):S410.

3. Kopylov U, Verstockt B, Biedermann L, et al. Effectiveness and Safety of Vedolizumab in Anti-TNF-Naïve Patients With Inflammatory Bowel Disease-A Multicenter Retrospective European Study. *Inflamm Bowel Dis*. 2018;24(11):2442-2451.

4. Ladd A, Scott F, Grace R, Bownik H, Lichtenstein G. Sa1086 Dose Escalation of Vedolizumab From Every 8 Weeks to Every 4 or 6 Weeks Enables Patients With Inflammatory Bowel Disease to Recapture Response. *Gastroenterology*. 2016;150:S235-S236.

5. Samaan MA, Birdi S, Morales MS, et al. Effectiveness of vedolizumab dose intensification to achieve inflammatory bowel disease control in cases of suboptimal response. *Frontline Gastroenterol*. 2020;11(3):188-193.

6. Shalabi A, Banerjee, R., Padley, J., Appleby, R., Johnston, E., . P149 Therapeutic Drug Monitoring of Vedolizumab For Patients With Inflammatory Bowel Disease: Real World Experience. *Gut*. 2021;70((Suppl 1)).

7. Bamias G, Kokkotis G, Gizis M, et al. Predictors of Response to Vedolizumab in Patients with Ulcerative Colitis: Results from the Greek VEDO-IBD Cohort. *Dig Dis Sci*. 2021:1-11.

8. Attauabi M, Hoglund C, Fassov J, et al. Vedolizumab as first-line biological therapy in elderly patients and those with contraindications for anti-TNF therapy: a real-world, nationwide cohort of patients with inflammatory bowel diseases. *Scand J Gastroenterol*. 2021;56(9):1040-1048.

9. Chaparro M, Garre A, Ricart E, Iborra M. Short and long-term effectiveness and safety of vedolizumab in inflammatory bowel disease: results from the ENEIDA registry. 2018;48(8):839-851.

10. Shmidt E, Kochhar G, Hartke J, et al. Predictors and Management of Loss of Response to Vedolizumab in Inflammatory Bowel Disease. *Inflamm Bowel Dis*. 2018;24(11):2461-2467.

11. Shmidt E, Winters A, Katta L, et al. P-040 Assessing Risk Factors Predicting Loss of Response to Vedolizumab in Ulcerative Colitis and Crohn's Disease: Outcomes from the VICTORY Consortium. *Inflamm Bowel Dis*. 2017;23(suppl_1):S18-S18.

12. Dulai PS, Singh S, Jiang X, et al. The Real-World Effectiveness and Safety of Vedolizumab for Moderate-Severe Crohn's Disease: Results From the US VICTORY Consortium. *Am J Gastroenterol*. 2016;111(8):1147-55.

13. Lee SD, Singla, A., Kerwin, C., Clark-Snustad, K. P089 Real-World Experience: Clinical and Endoscopic Effectiveness of Standard Vedolizumab Dosing and Modified Maintenance Dosing in Patients With Moderate-Severe Crohn’s Disease. *Gastroenterology*. 2020;158(3S):122.

14. Lee SD, Singla, A., Clark-Snustad, K. Standard and modified vedolizumab dosing is effective in achieving clinical and endoscopic remission in moderate‐severe Crohn’s disease. *GastroHep*. 2021;3(1):26-36.

15. Zingone F, Barberio B, Compostella F, et al. Good efficacy and safety of vedolizumab in Crohn's disease and ulcerative colitis in a real-world scenario. *Therap Adv Gastroenterol*. 2020;13:1756284820936536.

16. Tessolini J BG, Zezos P, Silverberg MS. Maintenance levels of vedolizumab do not correlate with clinical remission in IBD patients. *United European Gastroenterology Journal*. 2018;11(4):A66-7.

17. Plevris N, Jones GR, Jenkinson PW, et al. P471 Association of vedolizumab levels with clinical and biochemical markers of inflammation during maintenance therapy in inflammatory bowel disease. *Journal of Crohn's and Colitis*. 2019;13(Supplement_1):S347-S347.

18. Al-Bawardy B, Loftus EV, Jr., Ramos GP, et al. P167 VEDOLIZUMAB TROUGH LEVELS AND ANTIBODIES IN INFLAMMATORY BOWEL DISEASE: UPDATED INITIAL EXPERIENCE. *Inflamm Bowel Dis*. 2018;24(suppl_1):S62-S63.

19. Al-Bawardy B, Ramos GP, Willrich MAV, et al. Vedolizumab Drug Level Correlation With Clinical Remission, Biomarker Normalization, and Mucosal Healing in Inflammatory Bowel Disease. *Inflamm Bowel Dis*. 2019;25(3):580-586.

20. Torres N, Martín Arranz D, Sánchez Azofra M, et al. P546 Therapeutic drug monitoring of vedolizumab in inflammatory bowel disease. *Journal of Crohn's and Colitis*. 2019;13(Supplement_1):S387-S387.

21. Ungaro RC, Yarur A, Jossen J, et al. Higher Trough Vedolizumab Concentrations During Maintenance Therapy are Associated With Corticosteroid-Free Remission in Inflammatory Bowel Disease. *J Crohns Colitis*. 2019;13(8):963-969.

22. Ansari M, Glassner, K., Irani, M., Perry, C., Abraham, B.P.,. S0843 Therapeutic Drug Monitoring in Inflammatory Bowel Disease Patients on Vedolizumab. . *Am J Gastroenterol*. 2020;115:S435-436.

23. Ritter T, Fourment C, Van Anglen L, Hardin T, Schroeder C. Long-term safety analysis of vedolizumab for treatment of Crohn's disease and ulcerative colitis. *American Journal of Gastroenterology*. 2017;112.

24. Meserve J, Aniwan S, Koliani-Pace JL, et al. Retrospective Analysis of Safety of Vedolizumab in Patients With Inflammatory Bowel Diseases. *Clin Gastroenterol Hepatol*. 2019;17(8):1533-1540.e2.

25. Dimopoulos C, Hung K, Proctor D, Ruggiero E, Al-Bawardy B. 23 EFFECTS OF VEDOLIZUMAB ON EXTRAINTESTINAL MANIFESTATIONS IN INFLAMMATORY BOWEL DISEASE. *Inflamm Bowel Dis*. 2020;26(Supplement_1):S19-S20.

26. Guidi L, Pugliese D. Early vedolizumab trough levels predict treatment persistence over the first year in inflammatory bowel disease. 2019;7(9):1189-1197.

27. Biemans VBC, van der Woude CJ, Dijkstra G, et al. Vedolizumab for Inflammatory Bowel Disease: Two-Year Results of the Initiative on Crohn and Colitis (ICC) Registry, A Nationwide Prospective Observational Cohort Study: ICC Registry - Vedolizumab. *Clin Pharmacol Ther*. 2020;107(5):1189-1199.

28. Amor M, Lobato-Matilla E, Giménez-Manzorro Á, et al. 5PSQ-071 Evaluation of the effectiveness and safety of vedolizumab for the treatment of inflammatory bowel disease. *European Journal of Hospital Pharmacy*. 2019;26(Suppl 1):A234-A234.

29. Graziano EJ, Gheysens K, Cross RK, Campbell J, Vaughn B. 666 Outcomes Following Therapeutic Drug Monitoring of Vedolizumab. *American Journal of Gastroenterology*. 2019;114:S390-S391.

30. Tessolini JM, Bajaj G, Zezos P, et al. Maintenance Levels of Vedolizumab Do Not Correlate with Clinical Remission in IBD Patients. *Gastroenterology*. 2019;156(6):S-1140.

31. Albshesh A, Taylor J, Savarino EV, et al. Effectiveness of Third-Class Biologic Treatment in Crohn's Disease: A Multi-Center Retrospective Cohort Study. *J Clin Med*. 2021;10(13).

32. Kolehmainen S, Ylisaukko-Oja T, Jokelainen J, Koivusalo M, Jokiranta TS, Sipponen T. Benefit of measuring vedolizumab concentrations in inflammatory bowel disease patients in a real-world setting. *Scand J Gastroenterol*. 2021;56(8):906-913.

33. Ungar B, Malickova K, Hanžel J, et al. Dose optimisation for Loss of Response to Vedolizumab- Pharmacokinetics and Immune Mechanisms. *J Crohns Colitis*. 2021;15(10):1707-1719.

34. Sierra Morales M, Birdi S, Samaan M, et al. P673 Vedolizumab dose escalation as a way of recapturing response in patients with inflammatory bowel disease. *Journal of Crohn's and Colitis*. 2018;12(supplement_1):S451-S452.

35. Wice M, Oppenheim S, Miller H, et al. Efficacy and safety of vedolizumab in patients with inflammatory bowel disease in a large tertiary medical center. *American Journal of Gastroenterology*. 2016;111.

36. Amiot A, Serrero M, Peyrin-Biroulet L, Filippi J, Pariente B, Roblin X. One-year effectiveness and safety of vedolizumab therapy for inflammatory bowel disease: a prospective multicentre cohort study. 2017;46(3):310-321.

37. Christensen B, Colman RJ, Micic D, et al. Vedolizumab as Induction and Maintenance for Inflammatory Bowel Disease: 12-month Effectiveness and Safety. *Inflamm Bowel Dis*. 2018;24(4):849-860.

38. Dreesen E, Verstockt B, Bian S, et al. Evidence to Support Monitoring of Vedolizumab Trough Concentrations in Patients With Inflammatory Bowel Diseases. *Clin Gastroenterol Hepatol*. 2018;16(12):1937-1946.e8.

39. Kopylov U, Avni-Biron I, Ron Y, et al. Effectiveness and safety of vedolizumab for maintenance treatment in inflammatory bowel disease-The Israeli real world experience. *Dig Liver Dis*. 2019;51(1):68-74.

40. Williet N, Boschetti G, Fovet M, et al. Association Between Low Trough Levels of Vedolizumab During Induction Therapy for Inflammatory Bowel Diseases and Need for Additional Doses Within 6 Months. *Clin Gastroenterol Hepatol*. 2017;15(11):1750-1757.e3.

41. Dragoni G, Bagnoli S, Le Grazie M, et al. Long-term efficacy and safety of vedolizumab in patients with inflammatory bowel diseases: A real-life experience from a tertiary referral center. *J Dig Dis*. 2019;20(5):235-242.

42. Outtier AW, L. Rahier J-F, et al. Effect of vedolizumab dose intensification on serum drug concentrations and regain of response in inflammatory bowel disease patients with secondary loss of response. *GastroHep*. 2021;3(2):63-71.

43. Attauabi M, Vind I, Pedersen G, Bendtsen F, Seidelin JB, Burisch J. Short and long-term effectiveness and safety of vedolizumab in treatment-refractory patients with ulcerative colitis and Crohn's disease - a real-world two-center cohort study. *Eur J Gastroenterol Hepatol*. 2021.

44. Perry C, Fischer K, Elmoursi A, et al. Vedolizumab Dose Escalation Improves Therapeutic Response in a Subset of Patients with Ulcerative Colitis. *Dig Dis Sci*. 2021;66(6):2051-2058.
